# Artéria Coronária Direita Intracavitária: Um Achado Incidental com Potenciais Implicações para Procedimentos Cardíacos Invasivos

**DOI:** 10.36660/abc.20210819

**Published:** 2022-05-04

**Authors:** Sara Cristina da Silva Borges, Catarina Isabel Ribeiro Carvalho, Miguel Eduardo Teixeira Moz Gonçalves, Ana Isabel Santos Baptista, José Ilídio Moreira

**Affiliations:** 1 Departamento de Cardiologia Centro Hospitalar de Trás os Montes e Alto Douro Vila Real Portugal Departamento de Cardiologia, Centro Hospitalar de Trás os Montes e Alto Douro, Vila Real – Portugal

**Keywords:** Anormalidades Cardiovasculares, Anomalias dos Vasos Coronários, Angiografia Coronária/métodos, Angiotomografia do Coração, Diagnóstico por Imagem, Anomalias Intracavitárias/diagnóstico

Um homem de 66 anos com história de palpitações sugestivas de taquicardia supraventricular paroxística foi encaminhado para angiotomografia (ATC) para investigação da etiologia da dispneia de esforço. A TC cardíaca controlada por ECG foi realizada usando o Somatom Go Scanner de fonte dupla de 64 cortes.

**Figura 1:**
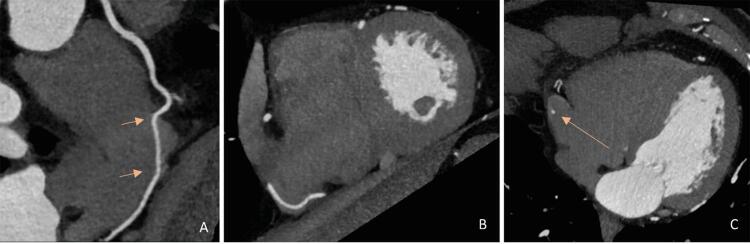
– Painel A) Imagem multiplanar curva mostrando o trajeto intra-atrial da artéria coronária direita (CD) (seta); Painel B) imagem de projeção de máxima intensidade mostrando a localização intra-atrial da CD; Painel C) Imagem axial de TC do trajeto intra-atrial da CD (seta).

**Figura 2:**
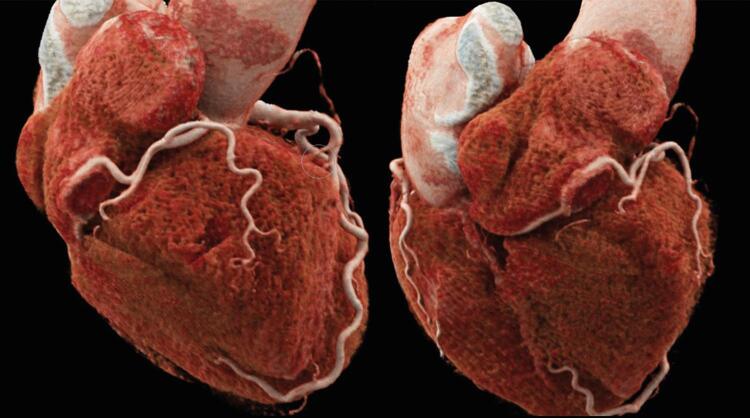
– Imagem 3D de angiotomografia coronariana mostra o trajeto epicárdico normal da CD proximal e sua entrada pela parede atrial direita.

A ATC mostrou a origem normal dos troncos das coronárias direita e esquerda, e não havia evidência de doença arterial coronariana obstrutiva. A artéria coronária direita proximal (CD) tinha um trajeto epicárdico normal, mas notou-se que a mediana penetrava na parede atrial direita por um trajeto de 30 mm dentro do átrio direito, saindo para seu trajeto usual no sulco atrioventricular posterior, conforme demonstrado via as imagens de TC de reconstrução multiplanar na projeção de intensidade máxima ( Figura 1 ), bem como as reconstruções tridimensionais ( Figura 2 ).

As anomalias das artérias coronárias (AACs) são definidas como um grupo de doenças congênitas caracterizadas por uma origem ou curso anormal de uma das principais artérias coronárias,com incidência variando de 1% a 5,6%.^1^ As variantes conhecidas do trajeto da artéria coronária podem ser amplamente classificadas em trajetos intramural, intracavitário e aéreo.

A ponte miocárdica é a presença de um trajeto intramural e é mais comumente reconhecida no segmento médio da descendente anterior esquerda (DAE). Os estudos mais recentes baseados em dados de ATC relatam uma prevalência de até 30%. Por outro lado, a artéria coronária intracavitária é uma rara variação anatômica isolada com duas variantes descritas – um trajeto intracavitário dentro da

artéria descendente anterior distal para o ventrículo direito e um trajeto intracavitário na ACD média para distal no átrio direito. Esta última é mais comum, com prevalência estimada de 0,36%,^2^ e cada vez mais reconhecida devido ao amplo uso de imagens cardíacas avançadas. A ATC é reconhecida como a técnica padrão-ouro para a avaliação de anomalias coronarianas congênitas, pois oferece os benefícios de imagens não invasivas de alta qualidade, exposição à radiação de baixa dose e oferece uma caracterização anatômica detalhada da origem e curso das artérias coronárias e sua relação com as estruturas circundantes.^2^

Embora geralmente clinicamente benigna e provavelmente não relacionada aos sintomas do nosso paciente, essa variante pode resultar em maior risco de dano inadvertido da ACD durante a manipulação do cateter no átrio direito.^2 - 4^

Em conclusão, identificar e descrever esta anomalia fornece informações cruciais para o cardiologista intervencionista ou o cirurgião e deve ser prontamente destacada para evitar complicações.^5^
