# Supplementary material for: Artéria Coronária Direita Intracavitária: Um Achado Incidental com Potenciais Implicações para Procedimentos Cardíacos Invasivos
Source: Arq Bras Cardiol. 2022 May 4;118(5):1000–1. [Article in Portuguese] doi: 10.36660/abc.20210819 (PMC9368867; doi:10.36660/abc.20210819)
Supplement: Translation

# Translation: Intracavitary Right Coronary Artery: An Incidental Finding with Potential Implications for Invasive Cardiac Procedures

**Authors:** Sara Cristina da Silva Borges, Catarina Isabel Ribeiro Carvalho, Miguel Eduardo Teixeira Moz Gonçalves, Ana Isabel Santos Baptista, José Ilídio Moreira

**Affiliations:** 1 Portugal Departamento de Cardiologia, Centro Hospitalar de Trás os Montes e Alto Douro, Vila Real – Portugal

**Keywords:** Cardiovascular Abnormalities, Coronary Vessel Anomalies, Coronary Angiography/methods, Tomography X-Ray Computed/methods, Anomalous Intracaitary /diagnosis

A 66-year-old man with a history of palpitations suggestive of paroxysmal supraventricular tachycardia was referred for CT angiography (CTA) for exertional dyspnea etiology investigation. ECG-gated cardiac CT was performed using the 64-slices dual-source Somatom Go Scanner.

CTA showed the normal origin of the right and left main coronary arteries, and there was no evidence of obstructive coronary artery disease. The proximal right coronary artery (RCA) had a normal epicardial course, but mid-RCA was noted to penetrate the right atrial wall for a 30 mm course within the right atrium, exiting to its usual course in the posterior atrioventricular groove, as demonstrated via the multiplanar reconstruction CT images at maximum intensity projection ( Figure 1 ) as well as 3-dimensional reconstructions ( Figure 2 ).

**Figure 1:** – Panel A) Curved multiplanar image showing the intra-atrial course of right coronary artery (RCA) (arrow); Panel B) maximum intensity projection image showing the intra-atrial location of the RCA; Panel C) Axial CT image of the intra-atrial course of RCA (arrow).

**Figure 2:** – CT coronary angiography 3D image shows the proximal RCA’s normal epicardial course and its entry through the right atrial wall.

Coronary artery anomalies (CAAs) are defined as a group of congenital disorders characterized by an abnormal origin or course of one of the main coronary arteries, with an incidence ranging from 1% to 5.6%.^1^ Known variants of a coronary artery trajectory can be broadly classified in intramural, intracavitary and aerial courses.

Myocardial bridging is a presence of an intramural course and is most commonly recognized in the middle segment of the left anterior descending (LAD). The most recent studies based on CTA data report a prevalence as high as 30%. On the other hand, the Intracavitary coronary artery is a rare isolated anatomic variation with two described variants – an intracavitary course within the distal left anterior descending artery into the right ventricle and an intracavitary course in mid to distal RCA into the right atrium. The latter is more common, with an estimated prevalence of 0.36%,^2^ and is increasingly recognized given the widespread use of advanced cardiac imaging. CTA is well recognized as the gold standard technique for the evaluation of congenital coronary anomalies as it provides the benefits of non-invasive high-quality imaging, low dose radiation exposure and offers a detailed anatomic characterization of origin and course of coronary arteries and its relationship with the surrounding structures.^2^

While usually clinically benign and probably unrelated to our patient’s symptoms, this variant may result in a higher risk of RCA inadvertent damage during catheter manipulation in the right atrium.^2 - 4^

In conclusion, identifying and describing this anomaly provides crucial information to the interventional cardiologist or surgeon and should be promptly highlighted in order to prevent complications.^5^
